# DEVELOPMENT AND VALIDATION OF A PSYCHOLOGICAL SCALE FOR BARIATRIC SURGERY: THE BARITEST

**DOI:** 10.1590/0102-672020220002e1682

**Published:** 2022-09-09

**Authors:** Carolina Mocellin Ghizoni, Fábio Brasil, César Augusto Taconeli, Lígia de Oliveira Carlos, Flávia Saboia, Giorgio Alfredo Pedroso Baretta, Magda Rosa Ramos da Cruz, Antônio Carlos Ligocki Campos

**Affiliations:** 1 Universidade Federal do Paraná, Surgical Clinic – Curitiba (PR), Brazil; 2 Universidade Federal do Paraná, Pharmaceutical Sciences – Curitiba (PR), Brazil; 3 Universidade Federal do Paraná, Statistic – Curitiba (PR), Brazil; 4 Prometheus Institute, Analytical Psychology – Maringá (PR), Brazil.

**Keywords:** Bariatric Surgery, Psychometrics, Psychological Tests, Obesity, Cirurgia Bariátrica, Psicometria, Testes Psicológicos, Obesidade

## Abstract

**BACKGROUND:**

It is recommended that bariatric surgery candidates undergo psychological assessment. However, no specific instrument exists to assess the psychological well-being of bariatric patients, before and after surgery, and for which all constructs are valid for both genders.

**AIMS:**

This study aimed to develop and validate a new psychometric instrument to be used before and after bariatric surgery in order to assess psychological outcomes of patients.

**METHODS:**

This is a cross-sectional study that composed of 660 individuals from the community and bariatric patients. BariTest was developed on a Likert scale consisting of 59 items, distributed in 6 constructs, which assess the psychological well-being that influences bariatric surgery: emotional state, eating behavior, quality of life, relationship with body weight, alcohol consumption, and social support. Validation of BariTest was developed by the confirmatory factor analysis to check the content, criteria, and construct. The R statistical software version 3.5.0 was used in all analyses, and a significance level of 5% was used.

**RESULTS:**

Adjusted indices of the confirmatory factor analysis model indicate adequate adjustment. Cronbach’s alpha of BariTest was 0.93, which indicates good internal consistency. The scores of the emotional state, eating behavior, and quality of life constructs were similar between the results obtained in the community and in the postoperative group, being higher than in the preoperative group. Alcohol consumption was similar in the preoperative and postoperative groups and was lower than the community group.

**CONCLUSIONS:**

BariTest is a reliable scale measuring the psychological well-being of patients either before or after bariatric surgery.

## RESUMO

**RACIONAL:**

Recomenda-se que os candidatos à cirurgia bariátrica sejam submetidos a uma avaliação psicológica. Contudo, não existe nenhum instrumento específico para avaliar os pacientes bariátricos, e que todos os construtos sejam válidos para ambos os sexos.

**OBJETIVOS:**

Desenvolver e validar um novo instrumento psicométrico para avaliar o bem-estar psicológico dos pacientes antes e após a cirurgia bariátrica.

**MÉTODOS:**

O estudo foi transversal e composto por 660 indivíduos da comunidade e pacientes bariátricos. O BariTest foi desenvolvido numa escala Likert composta por 59 itens, distribuídos em seis construtos, que avaliam o bem-estar psicológico que influenciam a cirurgia bariátrica: estado emocional, comportamento alimentar, qualidade de vida, relação com o peso corporal, consumo de álcool, e suporte social. A validação do BariTest foi feita pela validação de conteúdo, critério e construto e utilizou-se análise fatorial confirmatória. O software estatístico R versão 3.5.0, foi utilizado em todas as análises, com um nível de significância de 5%.

**RESULTADOS:**

Os índices ajustados do modelo análise fatorial confirmatória indicam um ajustamento adequado. O alfa de Cronbach do BariTest foi 0,93, o que indica uma boa consistência interna. As pontuações de estado emocional, comportamento alimentar e qualidade de vida foram semelhantes na comunidade e no grupo pós-operatório, sendo mais elevados do que no grupo pré-operatório. O consumo de álcool foi semelhante nos grupos pré e pós-operatórios e foi inferior ao do grupo comunitário.

**CONCLUSÕES:**

O BariTest é uma escala confiável que mede o bem-estar psicológico dos pacientes antes e após a cirurgia bariátrica.

## INTRODUCTION

Obesity is a chronic disease of multifactorial causes such as genetic, environmental, socioeconomic, endocrine, metabolic, and psychiatric^17^. When conventional treatments such as diet, medication, and physical exercise do not show any positive results and that obesity causes harm to the individual, bariatric surgery may be recommended^25^.

The candidates for bariatric surgery must have a body mass index (BMI) above 35 associated with a comorbidity (e.g., high blood pressure, diabetes, and hepatic steatosis, among others mentioned in Resolution No. 2,131/15 of the Federal Council of Medicine)^9^ or a BMI above 40, considered morbidly obese. The American Society for Metabolic and Bariatric Surgery (ASMBS)^1^ recommends that the candidates for bariatric surgery be followed up by a multidisciplinary team. In this team, the psychologist’s objective is to assess the candidate’s mental aptitude in order to understand the surgical procedure and the psychological aspects that can influence the result of the operation^38,42^.

Wadden and Sarwer^46^ suggested that in the psychological evaluation process, 70–90% of patients are unconditionally indicated for surgery, 15–30% are referred for psychological or nutritional treatment as a prerequisite for surgery, and the remaining patients are excluded due to psychiatric reasons such as psychosis, untreated severe depression, mood disorders, eating disorders, substance use disorder, psychosocial problems, or behavioral noncompliance.

Psychological treatment should be started in the preoperative phase because the candidates for bariatric surgery have a higher prevalence of mental disorders than the general population, and psychopathological abnormalities tend to impact both the evolution of obesity and the results of bariatric surgery^29,39^. Caution is recommended to indicate bariatric surgery in patients with severe psychiatric disorders without treatment. This is suggested when there is an absence of social support in those who, due to emotional instability, may find it difficult to follow and obey postoperative dietary instructions, and in cases of abuse of illicit drugs and/or alcoholism^9,41^.

A difficulty that professionals who make psychological assessment for bariatric surgery face is the lack of specific validated instruments for this population^22^. Psychologists vary in their methods of evaluating patients before and after bariatric surgery^10^. They usually apply symptom inventories to screen for depression and eating disorders, and some psychopathology, personality, or cognitive function tests^46^. The most cited assessment instruments in the literature^16^ are the Beck Depression Inventory (BDI), the Binge Eating Scale (BES), the Eating Disorder Examination, the Millon Behavioral Medicine Diagnostic (MBMD), and the Minnesota Multiphasic Personality Inventory (MMPI). These instruments were not developed with a focus on the bariatric population and the psychologist should avoid using several instruments because the patient’s tiredness may interfere in the accuracy of the answers^20^. Among the instruments intended for bariatric surgery, there is only one psychological instrument validated for the bariatric population, i.e., the PsyBari, developed by David Mahony, PhD, a clinical psychologist at the Lutheran Medical Center, Brooklyn, New York^27^. Despite being practical on a Likert scale and intended to assess bariatric patients before bariatric surgery, not all items of the test were valid for both genders. This is an important characteristic as there are two different test formats for each gender and it is questioned whether it is a single instrument or whether there are two distinct instruments, bringing unnecessary complexity. In addition, PsyBari validation was not performed with post-bariatric patients, and it is known that there is a significantly higher prevalence of alcohol consumption after bariatric surgery^23^, and some patients have an aggravation of the psychiatric disorder, which may worsen the patient’s psychological well-being, despite weight loss^38^. Furthermore, it is important to continue the psychological follow-up after bariatric surgery because some patients do not have a favorable outcome, which can lead to depression, use of alcoholic beverages, and weight regain^8,32^. Between 20 and 30% of patients experience suboptimal weight loss or significant weight regain within the first few postoperative years. The reasons for this involve physiological, behavioral, and psychological characteristics^38^.

Nowadays, no psychometric scale has been identified for which all of the instrument assesses both genders, before and after surgery, regardless of the surgical technique, focusing to assess the psychological well-being that can influence the outcome of the operation, such as severe depression, mood disorders, substance use disorder, eating disorders, psychosocial problems, or behavioral noncompliance^38^. Considering the six main psychological aspects that can influence the result of the operation^38^, BariTest was developed to compare the outcomes of psychological well-being that will emerge from bariatric surgery^20,38^.

The BariTest is a patient-reported outcome measures (PROM) psychometric scale which assesses the psychological well-being, before and after the bariatric surgery^9,38,42^, and is composed of six constructs:

1. emotional state;
2. eating behavior;
3. alcohol consumption;
4. social support;
5. relationship with body weight;
6. quality of life

These constructs are represented in 59 items answered by PROM, on a four-point Likert scale: 0= Never, 1= Rarely, 2= Sometimes, 3= Often, 4= Always (Table 1). The preparation and validation of BariTest was carried out through content, construct, and criterion validity, as suggested by Erthal^15^, Hutz^20^, Pasquali^35^, and American Educational Research Association^1^.

**Table 1:** BariTest: psychometric scale to bariatric patients.

BariTest It is important that you answer all items, putting the answer that you most identify with at this moment.		0 NEVER	1 RARELY	2 SOMETIMES	3 FREQUENTLY	4 ALWAYS
1	There are days when I feel a tightness in my chest, as if I am distressed.					
2	There are times when I cry a lot.					
3	I find myself in a bad mood and/or irritated for no reason.					
4	There are days when I wake up extremely excited and others, I hardly want to get out of bed.					
5	There are times when I feel like dying.					
6	I believe that I do things impulsively.					
7	People say that I am anxious.					
8	I have difficulty falling asleep because I feel very agitated and/or with rapid thoughts at night.					
9	I do and/or say things without thinking.					
10	I feel discouraged and hopeless.					
11	I have bouts of tachycardia, despair, and the feeling that I am going to die.					
12	I have a feeling of regret for the things I do/say.					
13	I believe that I am a disappointment for my family and/or friends.					
14	There are phases that I work too much and produce a lot, and in other phases, I don’t feel like working, and my work doesn’t produce.					
15	I realize that I talk too much or speak much faster than normal.					
16	When I’m eating, I lose control and end up eating too much.					
17	When I feel the urge to eat it is difficult to control.					
18	When I feel like eating some treats, I cannot put it off.					
19	I eat a few times a day, but when I eat, I exaggerate the quantity.					
20	When I have emotional problems, I use food to relieve tension or to bring me joy.					
21	I have a habit of eating “fast food” (snacks).					
22	I eat quickly and chew food sparingly.					
23	I think about food most of the day.					
24	I am a candy eater.					
25	My behavior toward food causes me a lot of suffering.					
26	I realize that I eat more at night.					
27	I have difficulty in distinguishing between hunger and the desire to eat.					
28	I eat sparingly in front of others, but then I make up for it when I’m alone.					
29	I eat small amounts of food for several hours in a row (Pinch Habit).					
30	I have a habit of eating when distracted by the TV, cell phone, computer, …					
31	I have difficulty leaving food on the plate at the end of a meal.					
32*	I feel supported and valued as a person.					
33*	I like the way I relate to people.					
34*	I consider myself an optimistic person and I have positive thoughts.					
35*	I am satisfied with my sex life.					
36*	I perform physical activity.					
37*	I perform leisure activities.					
38	I feel pain in my body.					
39*	I believe I have quality of life.					
40*	I have quality sleep.					
41	I stop going to social environments because of my physical appearance.					
42	I feel ashamed because of my weight.					
43	I believe I have problems at work because of my weight.					
44	I believe that people who live with me would love me more if I were thinner.					
45	I have difficulty performing my personal hygiene because of my weight.					
46	I avoid places until I know if there will be a place where I can sit.					
47	The next morning, after drinking, I wake up with a hangover. (If you don’t drink, mark with 0).					
48	I am in the habit of using alcohol to relax and be happy.					
49	People tell me that I am drinking too much.					
50	I have already cancelled appointments due to drinking the day before.					
51	I don’t like going to social events that don’t have alcohol.					
52	I notice that my family/friends insist that I eat more.					
53	I believe that my family/friends are offended if I refuse any food.					
54	In my family, people are in the habit of eating (includes meals/snacks/sweets) in front of the TV.					
55*	My family has a healthy lifestyle (food and physical activity).					
56*	I have family/friends support to facilitate my health care (e.g., taking care of children when I have an appointment, taking care of the house when I need help, …).					
57*	My family members acquired a healthier lifestyle to help me lose weight.					
58*	I believe I have people with whom I can vent or talk about issues related to my health, obesity, and/or weight loss.					
59*	I am satisfied with the support I receive from my friends/family.					

## METHODS

### Participants

This is a cross-sectional BariTest validation study, approved by the Research Ethics Committee of the Pontifícia Universidade Católica do Paraná, Curitiba, PR, Brazil, under number CAAE: 12476019.3.0000.0020. This study involved 660 people. Of these, 598 were awaiting consultation (preoperative or postoperative) at the bariatric surgery. In addition, for validation purposes, BariTest was applied to 48 nonobese subjects in the community, who had not undergone and did not intend to undergo bariatric surgery (Table 2). The instrument was also evaluated by a focus group (validity of content), selected as a convenience sample, composed of 10 bariatric patients who analyzed the semantic understanding of the item. Four patients did not respond to the questionnaire and were excluded from the analysis.

**Table 2:** Sociodemographic data of the participants in the BariTest validation.

Group	Characteristic	Group 1 – before bariatric (n=464)		Group 2 – after bariatric (n=134)		Group 3 – control (n=48)	
		n	Mean (±SD)	n	Mean (±SD)	n	Mean (±SD)
BMI	*	464	40.56 (±5.71)	134	31.36 (±6.58)	48	22.83 (±2.95)
		n	Percentage	n	Percentage	n	Percentage
Gender	Female	364	78.44	120	89.55	41	85.41
	Male	100	21.55	14	10.44	7	14.58
Age range (years)	18–30	147	31.68	31	23.13	25	52.08
	31–45	231	49.78	50	37.31	13	27.08
	46 or above	85	18.31	52	38.8	6	12.5
Marital status	Single	154	33.18	40	29.85	19	39.58
	Marriage	262	56.46	69	51.49	25	52.08
	Other	45	9.69	25	18.65	4	8.33
Education	Elementary school	37	7.97	27	20.14	1	2.08
	High school/Technical	171	36.85	77	57.46	1	2.08
	university/postgraduation	253	54.52	27	20.14	46	95.83
Surgery technique	RYGB	335	72.19	126	94.02	*	*
	SG	85	18.31	5	3.73	*	*

Note:

Sociodemographic data. Cross-sectional study, therefore the participants in each group are different (n=646). Group 1 are patients who were in the preoperative period of bariatric surgery. Group 2 are postoperative patients. Group 3 are community. SD: standard deviation; BMI: body mass index; Other: separated, divorced, widowed; RYGB: gastric bypass surgical technique; SG: gastric sleeve surgical technique.

* Does not apply to this group.

### Validation of BariTest

The BariTest validation process was carried out through content, construct, and criterion validity. In addition, the instrument’s reliability was analyzed, and the instrument’s correction and interpretation table was elaborated.

After conducting a literature review and expert discussions, a preliminary version of the BariTest scale was developed. BariTest items were prepared by the authors, based on tests and scales: Bipolar Depression Rating Scale (BDRS), Eating Attitudes Test (EAT-26), Binge Eating Scale (BES), BDI-II, BAI, BIS-11, AUDIT, SF-36, World Health Organization Disability Assessment Schedule 2.0 (WHODAS 2.0), HADS, ETC-R, the Eating Disorder Examination, and the MMPI.

Initially, the instrument had 99 items. Content validity was performed by assessing seven specialists in bariatric surgery or psychology, and all items were evaluated (Appendix 1). The anonymity of the evaluators was maintained, and each committee member individually determined their agreement on whether each item should remain in BariTest, using a four-point Likert scale: 0= Very Bad, 1= Bad, 2= More or less, 3= Good, 4= Great. At the end of this assessment, the experts carried out a qualitative analysis and offered suggestions for improvements. Items that had a mean of less than 3.5, or that were considered irrelevant to the objective by at least two members of the expert committee, were removed from the instrument (Appendix 3). Thus after this analysis, 40 items were excluded and BariTest completed with 59 items (Table 1). Also a focal group analyzed the understanding of each item, and no items were excluded by this group.

The validity of construct was performed by confirmatory factor analysis (CFA) (Appendix 2). The fitted CFA model was evaluated through the indices^6,7,18,43,47^ such as standardized root mean squared residual (SRMR), root mean of the squares of the errors of approximation (RMSEA), comparative fit index (CFI), and Tucker-Lewis index (TLI).

The validity of criterion was performed to ascertain the accuracy of the instrument, by means of stability in equivalent forms of different tests^44^. To determine responsiveness, an analysis of the receiving operating characteristic (ROC) curve was performed, verifying accuracy through sensitivity and specificity (Figure 2). At the time of applying BariTest, 175 patients also received two other questionnaires: the WHODAS 2.0 (Annex 1), which is a self-administered questionnaire that measures functionality and disability related to any disease or health status, avoiding the researcher’s bias, and the Obesity — related Problems Scale (OP) (Annex 2), which is a scale of outcomes reported by patients that measures the impact of excess weight on psychosocial functioning. These instruments were chosen because they have been validated^4,5^ for the Brazilian population with obesity to measure psychological well-being.

### Reliability

Reliability was calculated using the instrument’s internal consistency. Cronbach’s alpha^3^ was calculated for the six dimensions of BariTest, assessed in four situations, i.e., considering the entire sample, only patients in the preoperative period, only in the postoperative period, and separating by gender (Table 3).

**Table 3:** Reliability of BariTest’s items, considering the entire bariatric sample and separating by gender.

Construct	Number of items	Bariatric sample (n=598)	Male (n=114)	Female (n=484)
		(95%CI)		
Emotional state	15	0.89 (0.88–0.90)	0.879	0.891
Eating behavior	16	0.91 (0.90–0.92)	0.904	0.915
Quality of life	9	0.75 (0.72–0.78)	0.793	0.736
Relationship with body weight	6	0.78 (0.75–0.80)	0.812	0.778
Alcohol consumption	5	0.85 (0.83–0.87)	0.842	0.844
Social support	8	0.62 (0.58–0.66)	0.613	0.628

CI: confidence interval.

### Standardization of BariTest

To correct BariTest, it was necessary to multiply the response of each item by its respective general BariTest coefficient (Appendix 2) and calculate the average. The factorial loads were previously staggered so that each patient achieved a minimum of zero and a maximum of 100 points. It is important to note that some items had the score reversed; thus, items 32, 33, 34, 35, 36, 37, 39, 40, 55, 56, 57, 58, and 59 had the inverted correction, whereby 4=0, 3=1, 2=2, 1=3, and 0=4.

To interpret the score obtained, it was necessary to use the reference levels table (Appendix 3), calculated through the standard score (percentile). The characteristics of the patient were considered when they answered the BariTest (preoperative or postoperative phase, age, and gender) to check the percentile corresponding to that score. The purpose of this subdivision was to compare the score obtained with that of another similar subject^35^. The higher the score, the more the unwanted behaviors related to the construct.

### Data Analysis

The results were expressed as mean and standard deviation when the scores were normally distributed. Differences between groups were assessed using the t- or F-test when the normality assumption holds, and the Mann-Whitney or Kruskal-Wallis test, otherwise. CFA was performed based on polychoric correlations, since they are indicated^12^ instead of the usual Pearson linear correlations when data are expressed on an ordinal scale (Likert). In addition, data imputation based on proportional chance regression models was used to fill the missing values. Patients who did not respond to most questions were excluded from the analysis. All conclusions were based on a significance level of 5%. The statistical software R^36^ version 3.5.0 was used in all analyses. The Psych library^37^ was used to obtain the Cronbach’s alpha, while the Lavaan library^48^ was used for the CFA.

## RESULTS

### BariTest

The BariTest psychometric scale was elaborated (Table 1).

### Sociodemographic data

This is a cross-sectional study; therefore, the three groups are composed of different people (Table 2).

### Validity of BariTest

**Figure 1:**
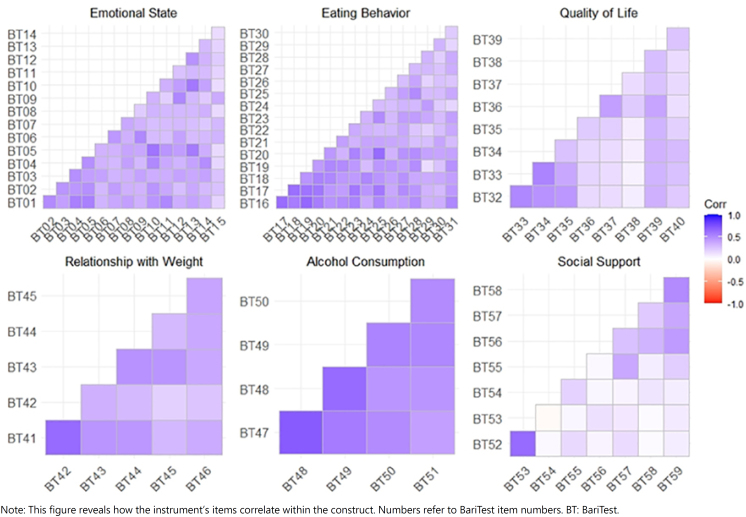
Correlations of the BariTest instrument items in their respective constructs.

For validation of BariTest, CFA (Appendix 2) was performed. The correlation between the items that make up each domain is shown in Figure 1. The factor loadings show how much the item is representative of construct. The more intense color tone shows a strong correlation; in contrast, the lower correlation level shows a weaker tone. The purple color represents a positive correlation, i.e., the answers point in the same direction, and the red represents a negative correlation, in which the answers point to the opposite of what that domain intends to prove. The variation ranges from 1 to −1, and the closer to 1 (purple color) means greater correlation between items. Therefore, the six BariTest factors show for the most part, strong and positive correlation.

The results of quality of the fit model are as follows: RMSEA of 0.064 (0.062; 0.066) and SRMR of 0.073 indicate an adequate fit, while the CFI of 0.926 and TLI of 0.923 indicate an acceptable fit^47^.

**Figure 2:**
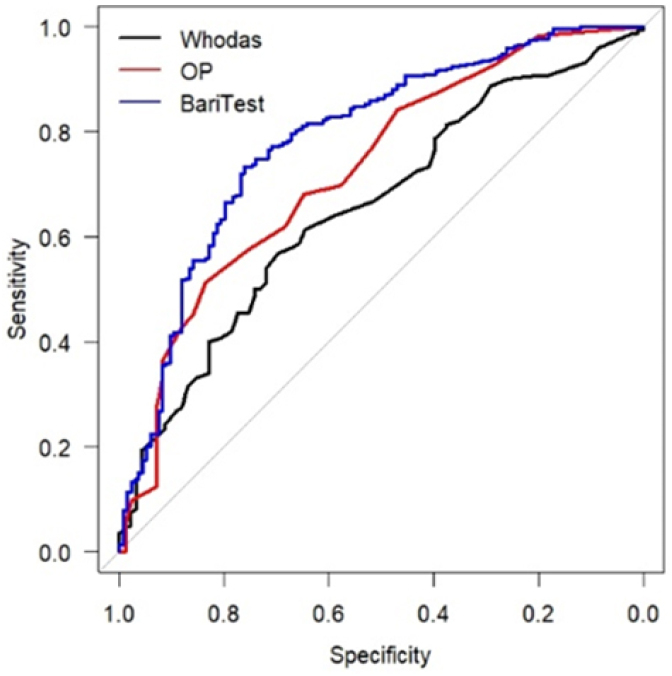
Illustration of BariTest’s responsivity (accuracy).

BariTest’s responsivity (accuracy) was verified in a comparative manner with the WHODAS 2.0 and OP scores (Annexes 1 and 2), by analysis of the areas under the ROC curves. Bariatric surgery causes changes in the psychological well-being of patients undergoing the procedure. The results showed that WHODAS 2.0 has 65% accuracy, OP has 72%, and BariTest has 78% (Figure 2), being. therefore, superior to the others to identify the chances of psychological well-being of the patient with obesity.

### Reliability

BariTest’s reliability showed a Cronbach’s alpha of 0.93 (95%CI, 0.92–0.94). The reliability of each construct was analyzed, considering the entire bariatric sample, and was separated by gender (Table 3). The similarity of the results showed^19^ that all of the instrument is valid for both genders.

### Results of BariTest

**Figure 3:**
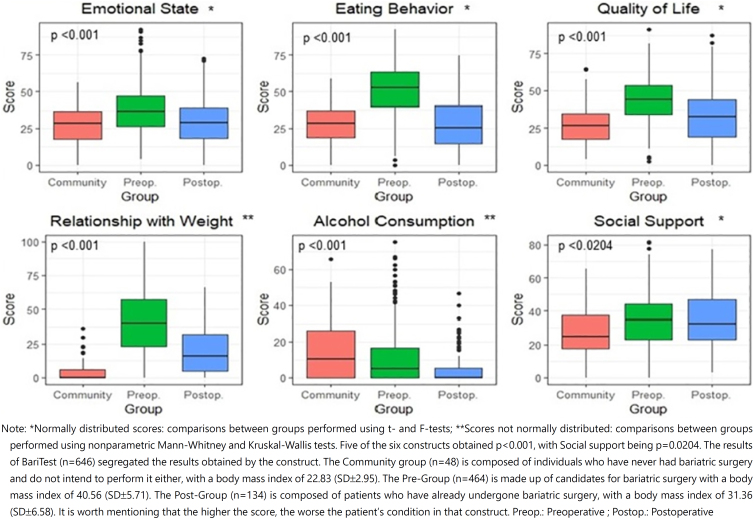
Comparison of the results of BariTest obtained between the preoperative, postoperative, and community.

The analysis between the constructs and groups (Figure 3) was adjusted for the results by the Bonferroni correction factor, to guarantee the significance level of 5%. The constructs Emotional state, Eating behavior, and Quality of life show a similarity between the results obtained in the community and postoperative groups and better than the preoperative group.

The community in general revealed to have more social support compared with obesity patients (preoperative and postoperative). Relationship with body weight differed in the three groups, possibly because the questions are specific to the bariatric population and the community was unable to answer.

Alcohol consumption was similar in the preoperative and postoperative groups and lower than the community group, indicating that people in the community consume more than the bariatric population. Five of the six constructs obtained p<0.001, with Social support being p=0.0204.

## DISCUSSION

There are numerous advantages for the psychologist to use BariTest, as it is a validated and complementary tool for psychological assessment that measures the psychological well-being of bariatric surgery patients. This instrument is valuable as a systematic procedure to collect, quantify, and evaluate the patient’s behavior and compare the psychological outcomes of the surgery^20,44^. The instrument was also applied in the community to nonobese subjects, with the sole purpose of verifying whether bariatric patients are distinct from the general population. Thus, BariTest proved that it is specific for the bariatric population, since the results obtained with candidates or patients who have already undergone bariatric surgery are different from the findings with the nonobese community.

The Emotional state construct consists of items that assess mood, anxiety, and impulsivity. Patients with obesity may have some cognitive difficulties, especially in the area of executive function responsible for planning, organizing, and controlling impulses^38,40^. The weight loss after bariatric surgery reduces neuroinflammation to rescue some aspects of defects in cognition and behavior^24^. Anxiety is the most common psychiatric disorder in patients with obesity who are awaiting bariatric surgery^14,21^.

The Emotional state score is similar between the postoperative period 29.7 (SD±16) and the community 27.1 (SD±13.8), but lower than the group that has not yet undergone surgery 37.8 (SD±15.5). This finding corroborates with the literature^11,31,32,34^ that shows the prevalence of depressive disorders being lower than in patients who have already undergone bariatric surgery and that patients who are in the preoperative period of bariatric surgery demonstrate more critical levels of depression, higher than those observed in the general population. In addition, worsening depression is associated with weight gain, which in turn leads to worse depression outcomes^2^.

The preoperative patients scored in BariTest’s Eating behavior (Figure 3), an average of 51.3 (SD±18.1), which was the highest average of all constructs, demonstrating that the candidate for bariatric surgery does not have a healthy relationship with food. It is important to assist the patient from the preoperative period, since studies have shown that the prevalence of binge eating symptoms in patients who are the candidates for bariatric surgery is 39–50% and is related to a suboptimal weight loss result after bariatric surgery^8,13,28,45^.

Quality of life and Relationship with body weight were constructs of BariTest which revealed a worse score in preoperative than postoperative and community. These data corroborate the prospective cross-sectional study by Moraes et al.^30^ who analyzed quality of life before and after bariatric surgery, reporting that 25% of patients considered quality of life and health to be poor or very bad before bariatric surgery, and after the procedure all patients rated it as good or very good.

The BariTest Social support construct revealed that bariatric patients (preoperative and postoperative) have less social support than the community and it is known that social support is associated with greater adherence to treatment and consequently successful outcomes^26^.

BariTest showed that the bariatric sample had an alcohol consumption lower than that of the general population. This finding was different from the study by King et al.^23^ and it is known that there is a significantly higher prevalence of alcohol consumption after bariatric surgery. It is believed that patients who are undergoing evaluation for bariatric surgery report a lower consumption of alcohol, since it is a contraindication for surgery. Furthermore, to have a low alcohol consumption in the postoperative period is important due to preventing alcoholism and weight regain^33^.

The results of the present study suggest that BariTest is a psychometric instrument capable of evaluating the psychological well-being of patients of both genders, before and after bariatric surgery (Table 3).

Even though BariTest has been validated with a significant number of patients, this study was cross sectional, because the aim of this study was to elaborate and validate this psychometric scale. Therefore after this stage, a longitudinal study would be very interesting to understand the changes that the surgery provides and perhaps predict the most suitable psychological profile for bariatric surgery. Sarwer et al.^38^ emphasize the importance of these studies to improve patient selection, improve psychoeducation and preoperative interventions, in addition to developing intervention strategies for patients who are unable to achieve the expected result after the procedure.

## CONCLUSION

BariTest is an instrument that makes it possible to measure and analyze psychological well-being and directs the necessary psychological interventions, before and after bariatric surgery, contributing to the psychological assessment. BariTest was developed as recommended in the scientific literature and proved all of the instrument was valid and reliable (α=0.93), measuring the psychological well-being of bariatric patients, regardless of gender, before and after bariatric surgery.

## Annex 1 Complementary Scales: WHODAS 2.0 – 36 items

WHODAS 2.0 WORLD HEALTH ORGANIZATION DISABILITY ASSESSMENT SCHEDULE 2.0

**36-item version, self-administered**

This questionnaire asks about difficulties due to health conditions. Health conditions include diseases or illnesses, other health problems that may be short or long lasting, injuries, mental or emotional problems, and problems with alcohol or drugs.

Think back over the past 30 days and answer these questions, thinking about how much difficulty you had doing the following activities. For each question, please circle only one response.

**Table:**

In the past 30 days, how much difficulty did you have in:							
Understanding and communicating							
	D1.1	Concentrating on doing something for ten minutes?	None	Mild	Moderate	Severe	Extreme or cannot do
	D1.2	Remembering to do important things?	None	Mild	Moderate	Severe	Extreme or cannot do
	D1.3	Analyzing and finding solutions to problems in day-to-day life?	None	Mild	Moderate	Severe	Extreme or cannot do
	D1.4	Learning a new task, for example, learning how to get to a new place?	None	Mild	Moderate	Severe	Extreme or cannot do
	D1.5	Generally understanding what people say?	None	Mild	Moderate	Severe	Extreme or cannot do
	D1.6	Starting and maintaining a conversation?	None	Mild	Moderate	Severe	Extreme or cannot do
Getting around							
	D2.1	Standing for long periods such as 30 min?	None	Mild	Moderate	Severe	Extreme or cannot do
	D2.2	Standing up from sitting down?	None	Mild	Moderate	Severe	Extreme or cannot do
	D2.3	Moving around inside your home?	None	Mild	Moderate	Severe	Extreme or cannot do
	D2.4	Getting out of your home?	None	Mild	Moderate	Severe	Extreme or cannot do
	D2.5	Walking a long distance such as a kilometer [or equivalent]?	None	Mild	Moderate	Severe	Extreme or cannot do

**Table:**

In the past 30 days, how much difficulty did you have in:							
Self-care							
	D3.1	Washing your whole body?	None	Mild	Moderate	Severe	Extreme or cannot do
	D3.2	Getting dressed?	None	Mild	Moderate	Severe	Extreme or cannot do
	D3.3	Eating?	None	Mild	Moderate	Severe	Extreme or cannot do
	D3.4	Staying by yourself for a few days?	None	Mild	Moderate	Severe	Extreme or cannot do
Getting along with people							
	D4.1	Dealing with people you do not know?	None	Mild	Moderate	Severe	Extreme or cannot do
	D4.2	Maintaining a friendship?	None	Mild	Moderate	Severe	Extreme or cannot do
	D4.3	Getting along with people who are close to you?	None	Mild	Moderate	Severe	Extreme or cannot do
	D4.4	Making new friends?	None	Mild	Moderate	Severe	Extreme or cannot do
	D4.5	Sexual activities?	None	Mild	Moderate	Severe	Extreme or cannot do
Life activities							
	D5.1	Taking care of your household responsibilities?	None	Mild	Moderate	Severe	Extreme or cannot do
	D5.2	Doing most important household tasks well?	None	Mild	Moderate	Severe	Extreme or cannot do
	D5.3	Getting all the household work done that you needed to do?	None	Mild	Moderate	Severe	Extreme or cannot do
	D5.4	Getting your household work done as quickly as needed?	None	Mild	Moderate	Severe	Extreme or cannot do

If you work (paid, non-paid, self-employed) or go to school, complete questions D5.5–D5.8, below. Otherwise, skip to D6.1.

**Table:**

Because of your health condition, in the past 30 days, how much difficulty did you have in:							
	D5.5	Your day-to-day work/school?	None	Mild	Moderate	Severe	Extreme or cannot do
	D5.6	Doing your most important work/school tasks well?	None	Mild	Moderate	Severe	Extreme or cannot do
	D5.7	Getting all the work done that you need to do?	None	Mild	Moderate	Severe	Extreme or cannot do
	D5.8	Getting your work done as quickly as needed?	None	Mild	Moderate	Severe	Extreme or cannot do

**Table:**

Participation in society							
In the past 30 days:							
	D6.1	How much of a problem did you have in joining in community activities (e.g., festivities, religious, or other activities) in the same way as anyone else can?	None	Mild	Moderate	Severe	Extreme or cannot do
	D6.2	How much of a problem did you have because of barriers or hindrances in the world around you?	None	Mild	Moderate	Severe	Extreme or cannot do
	D6.3	How much of a problem did you have living with dignity because of the attitudes and actions of others?	None	Mild	Moderate	Severe	Extreme or cannot do
	D6.4	How much time did you spend on your health condition, or its consequences?	None	Mild	Moderate	Severe	Extreme or cannot do
	D6.5	How much have you been emotionally affected by your health condition?	None	Mild	Moderate	Severe	Extreme or cannot do
	D6.6	How much has your health been a drain on the financial resources of you or your family?	None	Mild	Moderate	Severe	Extreme or cannot do
	D6.7	How much of a problem did your family have because of your health problems?	None	Mild	Moderate	Severe	Extreme or cannot do
	D6.8	How much of a problem did you have in doing things by yourself for relaxation or pleasure?	None	Mild	Moderate	Severe	Extreme or cannot do
	H1	Overall, in the past 30 days, how many days were these difficulties present?			**Record number of days ______**		
	H2	In the past 30 days, for how many days were you totally unable to carry out your usual activities or work because of any health condition?			**Record number of days ______**		
	H3	In the past 30 days, not counting the days that you were totally unable, for how many days did you cut back or reduce your usual activities or work because of any health condition?			**Record number of days ______**		

This completes the questionnaire. Thank you.

**Annex 2:** Complementary Scales: Brazilian version of the Obesity-related Problems Scale (OP) How do you feel about your weight or your body shape in the following situations?

OP1.	Receiving friends at home
OP2.	Visiting the home of relatives or friends
OP3.	Going to restaurants
OP4.	Doing activities in the community (courses etc.)
OP5.	Holidaying away from home
OP6.	Trying on and buying clothes
OP7.	Bathing in public places (beach, pool etc.)
OP8.	Intimate relationships (kiss, sex, etc.)

OP items are represented by the acronym “OP” followed by their ordering number.

All of them must be answered on a Likert scale as follows:

1. “It bothers me a lot.”
2. “It bothers me more or less.”
3. “It bothers me a little.”
4. “It doesn’t bother me.”

**Appendix 1:** Evaluation of the version of BARITEST by the Committee of Experts.

Items	Agreement index
1. When I’m eating, I feel like I’m losing control and I end up eating too much.	3.6
2. I think about food most of the day.	3.6
3. I eat sparingly in front of others, but then I make up for it when I’m alone.	4
4. I have a habit of eating when distracted by the TV, cell phone, computer.	3.6
5. I eat small amounts of food for several hours in a row (Pinch Habit).	3.7
6. When I feel sad or anxious or idle I have a habit of compensating with food and overeating.	3.7
7. I chew my food well and eat my meals calmly.	3.6
8. I eat few times a day, but when I eat, I overdo it.	3.6
9. I have difficulty in distinguishing between hunger and the desire to eat.	3.9
10. I have crises of eating a lot until I am full.	3.1
11. I have a habit of eating “fast food” (Snacks).	3.8
12. I am a candy eater.	3.5
13. When I feel the urge to eat, it is difficult to control myself.	3.9
14. I intend to eat just a little, but when I see it, I eat a lot more than I want to.	3.4
15. I notice that I eat more at night.	3.9
16. I wake up in the early hours to eat something.	3
17. When I go on a diet, I manage to stop eating some foods that I love, without any problem.	3.3
18. My behavior towards food causes me a lot of suffering.	3.9
19. When I have emotional problems, I use food to relieve tension or to bring me joy.	3.9
20. When I feel like eating a treat, I eat without delaying and/or depriving myself.	3.7
21. I have difficulty leaving food on the plate at the end of a meal.	3.9
22. The next morning, after drinking, I wake up with a hangover (If you don’t drink, mark 0).	3.6
23. I drink alcohol on weekends.	3.1
24. I drink alcohol during the week.	3.4
25. People tell me that I am drinking too much. (If you don’t drink, mark “never”).	3.5
26. I am in the habit of using alcohol to relax and be happy. (If you don’t drink, mark “never”).	3.9
27. After drinking alcohol, I missed or was late for an appointment the next day. (If you don’t drink, mark “never”).	3.6
28. I don’t like going to social events that don’t have alcohol.	3.9
29. I perform leisure activities.	3.7
30. I stop going to social settings (parties, meetings,.) due to my physical appearance or health limitations.	3.9
31. I believe I have problems with my work because of my weight.	3.6
32. I feel pain in my body.	3.6
33. I am satisfied with myself.	3.4
34. I feel happy.	3
35. I like the way I relate to people.	3.6
36. I am satisfied with my sex life.	3.5
37. I am satisfied with the support I receive from my friends/family.	3.6
38. I have negative feelings, such as: bad mood, despair, anxiety and/or depression.	3
39. I feel ashamed because of my weight.	3.5
40. I have quality sleep.	3.9
41. The physical environment (home or work) that I frequent is stressful (pollution, noise, traffic, arguing).	3.4
42. Religion is part of my life and/or I have a higher belief.	2.3
43. I find it difficult to perform my personal hygiene because of my weight.	3.5
44. I avoid places until I know if there will be a place where I can sit.	3.5
45. I believe I have quality of life.	3.9
46. I perform physical activity.	3.7
47. I find myself sulking and irritated for no reason.	3.5
48. There are times when I sleep a lot and times when I sleep little.	3
49. I believe I talk too much.	3.1
50. There are days when I wake up extremely excited and on others I barely feel like getting out of bed.	3.7
51. I feel very sad and/or unhappy.	3
52. I believe that there is nothing to achieve in my future.	3.1
53. I feel discouraged and hopeless.	3.5
54. I believe that I am a disappointment to my family and/or friends.	3.6
55. There are times when I feel like dying.	3.6
56. I think about ending my life.	3.3
57. There are times when I cry a lot.	3.6
58. I have or have had some type of auditory hallucination (heard voices).	2.7
59. I find myself much more interested in sex than usual.	3.1
60. There are phases that I work too much and produce a lot, and in other phases I don’t feel like working, and my work doesn’t produce.	3.5
61. I find that I get distracted or lose focus on what I’m doing very easily.	3.4
62. I feel that there are people following me and/or watching me.	3.1
63. I talk too much or speak much faster than normal.	3.5
64. Standing still causes me anxiety.	3.1
65. I have a feeling of regret for the things I do/say.	3.5
66. I feel so nervous that I have shortness of breath.	3.3
67. I have a tremor in my hands.	2.9
68. I feel more nervous than other people, with some everyday situations.	3.1
69. People say that I am anxious.	3.9
70. I find it difficult to fall asleep because I feel very agitated and/or with rapid thoughts at night.	3.6
71. I have bouts of tachycardia, despair and the feeling that I am going to die.	3.6
72. I believe that I do things impulsively.	3.9
73. I do and/or say things without thinking.	3.5
74. I can focus on just one thing for a long time.	3
75. I buy things on impulse, without really needing them.	3.4
76. There are situations where I think I’m going to lose control and go after someone.	3.4
77. I consider myself an optimistic person and I have positive thoughts.	3.8
78. I have self-control.	2.7
79. My family/friends are offended if I refuse any food.	3.6
80. My family/friends insist that I eat more.	3.5
81. In my family, people are in the habit of eating (includes meals, snacks and sweets) in front of the TV.	3.6
82. I have family/friends support to facilitate my health care (e.g., taking care of children when I have an appointment, taking care of the house when I need help).	4
83. My family has a healthy lifestyle (food and physical activity).	3.7
84. I feel supported and valued as a person.	3.9
85. I have people I can talk to or talk about issues related to my health, obesity and/or weight loss.	4
86. My family members acquired a healthier lifestyle to help me with the weight loss process.	3.5
87. I believe that the people who live with me would love me more if I were not obese.	3.5
88. I believe I have people with whom I can vent or talk about issues related to my health, obesity and/or weight loss.	3.7
89. I usually wait for things to work out over time.	2.9
90. I panic when difficulties arise.	2.9
91. To deal with difficulties, I make an action plan and try to apply it.	3.1
92. I know what I have to do and I redouble my efforts to achieve it.	3.4
93. I try to see the positive and/or make the best of situations.	3.2
94. When I have problems/difficulties, I face the situation.	3.3
95. I forget about my problems by denying and/or taking medication.	3.4
96. When I have a problem, I feel guilty.	2.9
97. When I have a problem, I distance myself from others.	3.2
98. When I have a problem, I don’t do anything, because I think I won’t be able to solve it.	3.1
99. I feel angry at the people who caused me a problem.	3.4

Version of BariTest with 99 items, assessed qualitatively and quantitatively (five-point Likert scale: 0= Very bad, 1= Bad, 2= More or less, 3= Good, 4= Great), by the expert committee. The questions that had an average below 3.5 or a critic in the qualitative analysis were removed from the instrument.

**Appendix 2:** Confirmatory factor analysis of BariTest.

Dimension	Item	Factorial loading	Standard error	Construct coefficient	BariTest general coefficient
Emotional state	1	10.000	0.0000	17.652	0.3273
	2	0.9204	0.0285	16.248	0.3013
	3	10.015	0.0298	17.678	0.3278
	4	10.849	0.0311	19.152	0.3551
	5	10.709	0.0309	18.904	0.3505
	6	0.9410	0.0288	16.611	0.3080
	7	0.8879	0.0280	15.674	0.2906
	8	0.8556	0.0275	15.103	0.2800
	9	0.8764	0.0278	15.471	0.2869
	10	11.378	0.0320	20.085	0.3724
	11	0.8203	0.0270	14.480	0.2685
	12	10.167	0.0300	17.947	0.3328
	13	10.668	0.0308	18.831	0.3492
	14	0.9734	0.0293	17.182	0.3186
	15	0.5087	0.0231	0.8980	0.1665
Eating behavior	16	10.000	0.0000	19.187	0.3638
	17	10.166	0.0253	19.505	0.3698
	18	0.9094	0.0238	17.449	0.3308
	19	0.8733	0.0233	16.757	0.3177
	20	10.719	0.0261	20.567	0.3899
	21	0.7578	0.0219	14.539	0.2756
	22	0.6526	0.0206	12.521	0.2374
	23	0.8907	0.0236	17.089	0.3240
	24	0.6337	0.0204	12.159	0.2305
	25	0.9807	0.0248	18.816	0.3567
	26	0.6276	0.0204	12.043	0.2283
	27	0.7742	0.0221	14.855	0.2816
	28	0.8943	0.0236	17.158	0.3253
	29	0.5849	0.0199	11.222	0.2127
	30	0.5985	0.0201	11.483	0.2177
	31	0.7635	0.0219	14.649	0.2777
Quality of life	32*	10.000	0.0000	35.401	0.5531
	33*	0.9617	0.0346	34.046	0.5320
	34*	10.095	0.0355	35.738	0.5584
	35*	0.8073	0.0317	28.578	0.4465
	36*	0.6471	0.0290	22.909	0.3579
	37*	0.4676	0.0266	16.555	0.2587
	38	0.6006	0.0283	21.261	0.3322
	39*	0.9824	0.0350	34.779	0.5434
	40*	0.5856	0.0281	20.731	0.3239
Relationship with body weight	41	10.000	0.0000	44.830	0.7285
	42	10.660	0.0336	47.791	0.7766
	43	0.8484	0.0298	38.032	0.6180
	44	10.146	0.0327	45.486	0.7391
	45	0.7663	0.0285	34.352	0.5582
	46	0.8813	0.0303	39.508	0.6420
Alcohol consumption	47	10.000	0.0000	52.100	0.9226
	48	0.9320	0.0465	48.556	0.8599
	49	11.566	0.0548	60.259	10.671
	50	0.9126	0.0459	47.546	0.8420
	51	0.7973	0.0422	41.539	0.7356
	52	10.000	0.0000	28.210	0.3644
Social support	53	11.017	0.0737	31.079	0.4014
	54	0.5297	0.0551	14.944	0.1930
	55*	12.046	0.0778	33.981	0.4389
	56*	10.015	0.0699	28.253	0.3649
	57*	0.9904	0.0695	27.938	0.3609
	58*	12.749	0.0806	35.966	0.4646
	59*	17.592	0.1017	49.628	0.6410

This table contains the confirmatory factor analysis with the factorial loading and standard error of each item of BariTest.

* Items 32, 33, 34, 35, 36, 37, 39, 40, 55, 56, 57, 58, and 59 had the score reversed so that all domains point in the same direction of assessing psychological well-being. Calculation for correction of BariTest obtained through confirmatory factor analysis.

**Appendix 3:** Reference levels for interpreting the BariTest result.

Percentile	Group								
	Postop: 18–30	Postop: 31–45	Postop: 46+	Preop: F:18–30	Preop: F:31–45	Preop: F:46+	Preop: M:18–30	Preop: M:31–45	Preop: M:46+
2.5	6.087	5.462	0.580	20.054	17.081	11.480	18.479	14.233	8.013
5	9.728	8.533	4.024	23.199	20.302	14.861	21.158	17.426	11.371
10	13.926	12.074	7.994	26.825	24.016	18.759	24.247	21.107	15.242
15	16.759	14.463	10.673	29.272	26.521	21.389	26.331	23.591	17.854
20	19.010	16.362	12.802	31.217	28.513	23.479	27.988	25.565	19.929
25	20.941	17.991	14.629	32.885	30.221	25.272	29.409	27.259	21.710
30	22.675	19.453	16.269	34.383	31.756	26.882	30.685	28.780	23.309
35	24.282	20.809	17.789	35.771	33.177	28.374	31.868	30.189	24.791
40	25.807	22.095	19.232	37.088	34.526	29.790	32.990	31.526	26.198
45	27.283	23.340	20.627	38.363	35.832	31.160	34.076	32.820	27.558
50	28.735	24.565	22.000	39.617	37.116	32.509	35.144	34.093	28.897
55	30.187	25.789	23.374	40.871	38.401	33.857	36.212	35.367	30.236
60	31.662	27.034	24.769	42.146	39.706	35.227	37.298	36.660	31.597
65	33.187	28.320	26.212	43.463	41.055	36.643	38.420	37.998	33.003
70	34.794	29.676	27.732	44.851	42.477	38.135	39.603	39.407	34.485
75	36.529	31.139	29.372	46.350	44.011	39.745	40.879	40.928	36.084
80	38.460	32.768	31.199	48.018	45.719	41.538	42.300	42.621	37.865
85	40.711	34.666	33.328	49.962	47.711	43.628	43.956	44.595	39.941
90	43.543	37.055	36.007	52.409	50.216	46.258	46.041	47.079	42.552
95	47.741	40.596	39.977	56.035	53.930	50.156	49.130	50.760	46.423
97.5	51.382	43.668	43.421	59.180	57.151	53.537	51.809	53.953	49.781

Preop.: preoperative; Postop.: postoperative; M: Male; F: Female.
